# A109 TRANEXAMIC ACID TO PREVENT BLEEDING AFTER ENDOSCOPIC RESECTION OF LARGE COLORECTAL POLYPS: A PILOT PROJECT

**DOI:** 10.1093/jcag/gwac036.109

**Published:** 2023-03-07

**Authors:** M Rai, L Hookey, R Bechara

**Affiliations:** Department of Medicine, Division of Gastroenterology, Queen's University, Kingston, Canada

## Abstract

**Background:**

Colonoscopy and polypectomy reduce colorectal cancer incidence and mortality, but is also associated with adverse events, including bleeding. Postpolypectomy delayed bleeding (PPDB) after EMR of large colorectal polyps (≥2cm) has an incidence of 2.6-9.7%. Tranexamic acid is a member of a class of drugs called antifibrinolytic agents. It reduces fibrinolysis by slowing down the conversion of plasminogen to plasmin, which may prevent bleeding.

**Purpose:**

The goal of this pilot study is to assess the feasibility of using tranexamic acid after EMR of large (≥2 cm) non-pedunculated colorectal polyps (LNPCPs) to prevent PPDB.

**Method:**

This was a single center feasibility study conducted at the Kingston Health Sciences Center from March 2021 to September 2021. Patients referred for removal of a ≥2cm LNPCP and those who were referred for a positive fecal immunochemical test were approached for consideration of inclusion. Patients with INR ≥ 1.5, platelets <50, higher risk of risk of thromboembolic events (atrial fibrillation on anticoagulation, history of stroke, TIA, pulmonary embolism, deep vein thrombosis hypercoagulable state, mechanical heart valve on anticoagulation, myocardial infarction in the last twelve months), pregnancy or undergoing ESD were not included.

Coagulation of submucosal vessels after polypectomy by snare tip coagulation or forceps was performed if thought necessary by the endoscopist. Clipping could be performed only where there was concern for perforation. Intraprocedural bleeding was recorded and managed at the discretion of the endoscopist.

After the procedure was completed, 1 gram of TXA in 100mL of normal saline (NS) was infused over a 10-minute interval. The participants received tranexamic acid 1 gram PO TID to be taken for 5 days after the procedure. A post procedure day 5, 14 and 30 phone call was conducted with participants to monitor study drug compliance and adverse events.

**Result(s):**

A total of 25 patients were enrolled with a mean polyp size of 3 cm. Baseline patient and polyp characteristics are presented in table 1. 90% of eligible patients approached consented to be in the study. Procedure details are presented in table 2. Intraprocedural bleeding occurred in 7 patients (28%) and all of these were treated with soft coagulation. 2 patients had clipping for muscle injury. All 25 patients received IV TXA post procedure. 16 patients (64%) took every dose of the prescribed pills. 21 patients (84%) took at least 80% of the prescribed TXA pills. 1 patient presented with post polypectomy bleeding. All patients completed the day 30 follow up phone call. There were no adverse events.

**Image:**

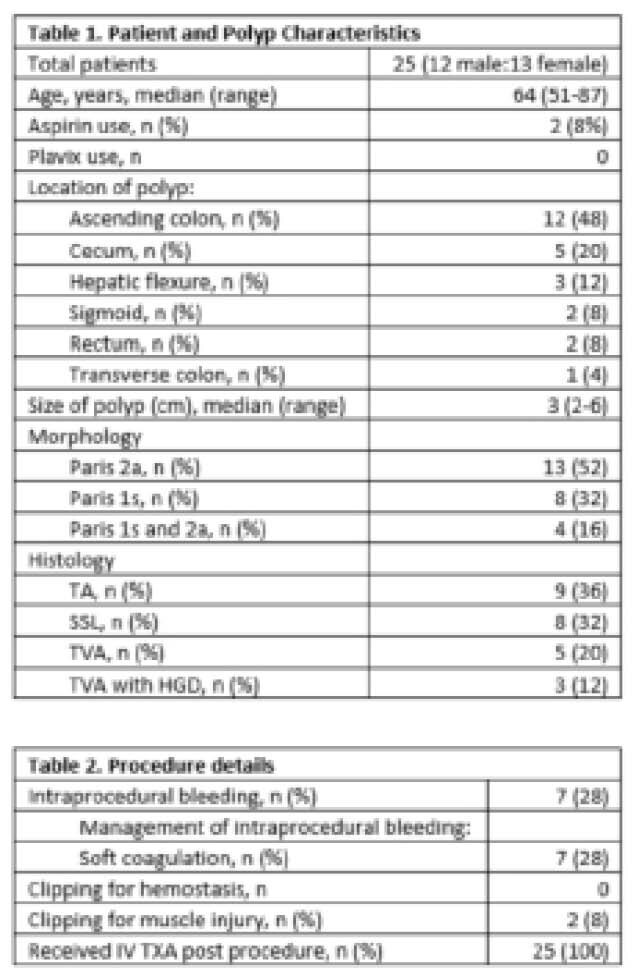

**Conclusion(s):**

TXA to prevent postpolypectomy delayed bleeding (PPDB) was feasible to use with no adverse events reported. All patients received IV TXA post procedure and completed 30 day follow up. However, only 64% of patients took every scheduled dose of medication. A randomized controlled study will be needed to see if TXA can significantly reduce PPDB.

**Please acknowledge all funding agencies by checking the applicable boxes below:**

Other

**Please indicate your source of funding;:**

Queen's University DOM Research Award – Clinical Innovation

**Disclosure of Interest:**

None Declared

